# Prediction of bolt supporting the controlling influence angle based on a synthetic weight factor

**DOI:** 10.1371/journal.pone.0276536

**Published:** 2022-10-27

**Authors:** Zhigao Liu, Shoubao Zhang, Weixiang Meng, Zhiliu Wang

**Affiliations:** 1 School of Energy and Mining Engineering, China University of Mining and Technology (Beijing), Beijing, China; 2 Taiyuan Iron & Steel (Group) CO. LTD, Taiyuan, China; 3 School of Civil Engineering and Architecture, Zhongyuan University of Technology, Zhengzhou, China; University of Vigo, SPAIN

## Abstract

The purpose of a rock bolt is to improve the strength capacity of a jointed rock mass. The strengthened arch controlling area can be formed based on the superposition of the controlling influence range of the bolt with the controlling influence angle of rock bolt playing an important role. However, quantitative research on the influence angle is still rare. In this study, numerical simulations and mathematical analysis are used to study the law of stress field distribution and the controlling influence angle through a single bolt, and the following conclusions can be obtained. (1) The compressive stress field is roughly distributed in an "Apple shape" and in a "conical" spatial distribution. (2) The bolt controlling angle is not a constant 45°, and it is influenced by the rock mass strength and bolt parameters. It decreases with the increasing elastic modulus of the bolt, bolt diameter and bolt length. It also increases with the increasing pretension and rock mass strength. The length has less influence on the supporting range. (3) Based on the experimental results, an optimal analytical model to predict the bolt’s controlling influence angle was developed. The analytical model includes the influences of the rock mass strength and bolt parameters. (4) A comparison between the model predictions with the results from the Dabei Mining 103 face transportation tunnel and the existing results shows the rationale behind the original support design scheme and an improvement over the existing results.

## 1. Introduction

As the most commonly used supporting material in underground coal mines, bolts have the advantages of low supporting costs, good supporting effects, and strong adaptability. One of the huge challenges that the bolting has to deal with is the protection of the stratified rock mass [1]. The bolt support can significantly control the generation and extension of joints. Furthermore, it can restrain the strain energy released due to the expansion of joint cracks [2].

The comprehensive stress field, including the in situ stress field, mining-induced stress field and supporting-induced stress field, are constituted [3,4]. Meanwhile, the traditional bolt supporting theory provides a more reasonable qualitative explanation for the bolt supporting mechanism, and the controlling influence angle of the rock bolt plays an important role in the bearing capacity of the tunnel [5–8]. Numerical simulation methods were used to conclude that the combined support of prestress bolts and cables can form mutually connected and superimposed effective compressive stress areas in the anchoring structure of the roadway [9,10]. A variety of analytical methods were proposed to reveal the large deformation mechanism under the superposition of multiple stress fields [11,12]. To study the physical-thermal coupling mechanism of anchoring, the deformation and failure processes of different types and infrared thermal imaging technology were used to obtain the infrared temperature field [13]. The concept of an "anchored composite bearing body" was put forward and preliminarily wrote the design software of roadway bolt supporting parameters [14]. Based on the numerical analysis software (PFC3D), a crushed stone anchorage experiment was carried out to reveal the formation mechanism and process of the pressure arch [15]. The supporting stress field was analysed and was composed of the prestress field generated by the bolt and the stress field released by the bolt restrained the deformation and failure of the surrounding rock [16]. Roadway support technology was elaborated on and analysed from the three aspects of surrounding rock, roadway surface and compound control, and some new technologies and new theories of roadway support were discussed [17–19]. Using the numerical simulation method, surrounding rock fissures were simulated, and the characteristics of bolt support were well studied [20]. A comprehensive and systematic study on the mechanical properties and mutual matching of each component of roadway bolt support was conducted [21,22]. Chen et al. [23,24] examined the effectiveness of different bolt anchoring systems in bedded rock to determine the bolt anchoring mechanism. Rock failure under shear and tensile stresses was evaluated. He et al [25,26] proposed a unified rock bolt model, and a numerical method was proposed to provide a detailed profile of the behaviour of bolts, particularly at intermediate loading stages. Chen et al. [27,28] studied the anchorage performance of bolt to different joint opening conditions. The results reveal that increasing the shearing strength of the grout/rock contact surface was conductive to improving the anchorage performance of bolts.

However, the bolt controlling influence angle (β) is generally considered to be 45° with relatively few quantitative studies on the β of a single bolt. The calculation of the anchoring bearing capacity is simplified, which leads to errors in the calculation results [29–32]. In fact, the research on β should be given a clearer explanation of the specific mechanism in the plastic zone, as it is very important for the reasonable selection of bolt parameters and the maintenance of the mutual coordination between the bolt and the cable.

In the following parts of the research, Section 2 presents the characteristics of the stress field of bolt support, the results of the numerical simulation, Section 3 describes and discusses the controlling influence angle law, the prediction model of the controlling influence angle is outlined, and the rationale behind the original support design in the Dabei Mining 103 face transportation tunnel is verified in Section 4. Finally, the findings and conclusions are presented in Section 5.

## 2. Stress characteristics and factors analysis of single rock bolt

The prestress bolt supporting system consists of a tray, a free section and an anchor section. The prestress applied by the bolt rod forms a compressive stress zone with the rock mass surface through the action of the bolt rod tray and spreads into the rock mass. Inside the rock mass, tensile stress is formed by the cement slurry between the inner anchor section and the rock mass. Numerical models of bolts under support were established here without considering the in situ stress field.

### 2.1 Analysis of the stress field distribution characteristics

**Fig 1:**
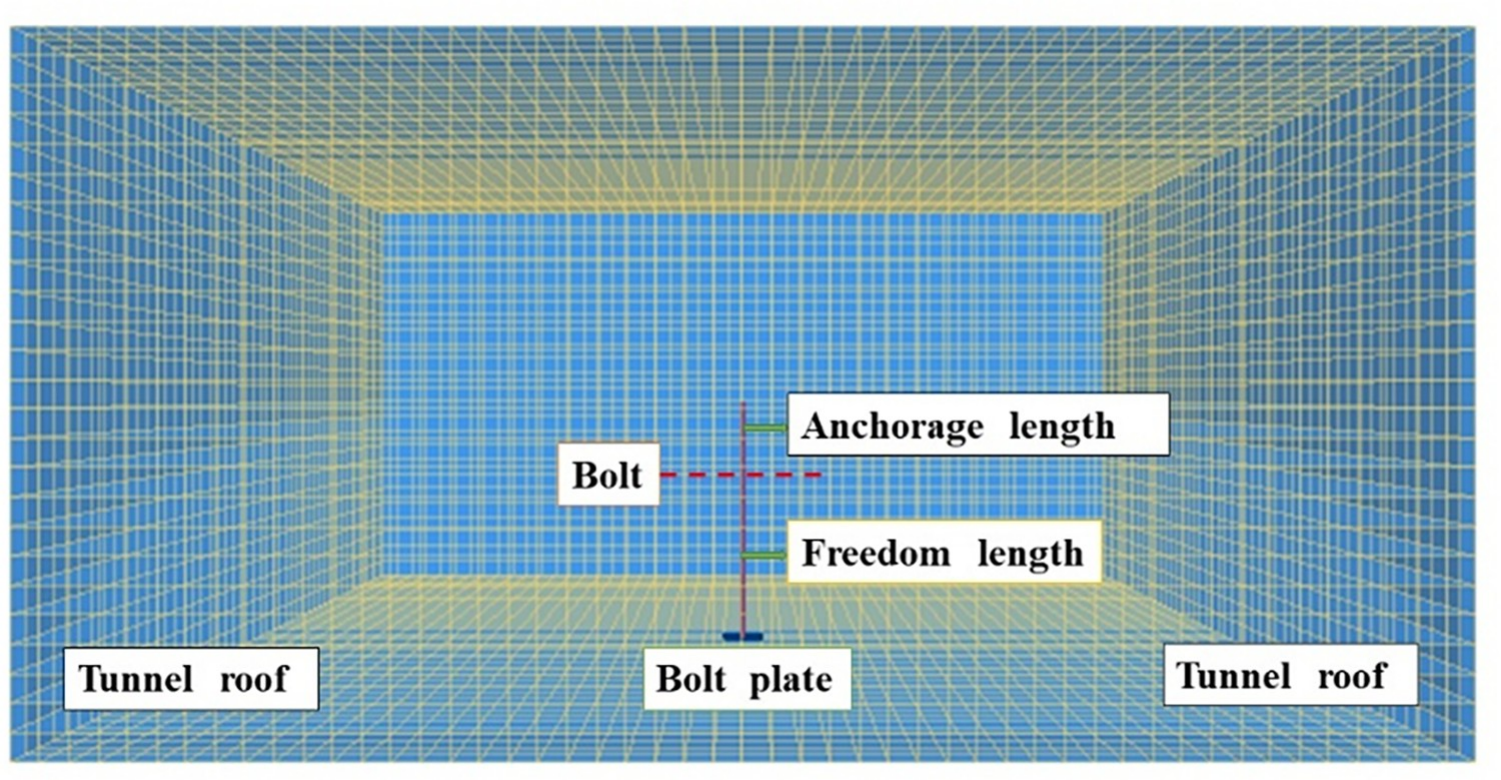
Bolt supporting model.

To understand the distribution characteristics more intuitively, FLAC^3D^ numerical simulation software is used, and the influence of the in situ stress field is temporarily ignored in this study. Based on the geological conditions of the engineering site, a single bolt supporting calculation model is established. The model of the solid anchor is 10 m×10 m×5 m. The block is divided into 62500 8-node hexahedron elements. The bolt is arranged at the position of the model where X = 5m, Y = 5m, and the anchor bolt length is 2.4 m (Fig 1). Mohr-Coulomb failure criterion is used for the numerical calculation and the rock mass-binder-bolt rod contact was simulated by elastic-perfectly plastic model.

The model consists of a kind of rock mass. The mechanical parameters of the rock mass and bolt are shown in Table 1.

**Table 1:** Mechanical parameters of rock mass.

**Rock property**	**Bulk modulus** **/GPa**	**Shear modulus** **/GPa**	**Friction** **/(°)**	**Uniaxial compressive strengh** **/MPa**	**Cohesion** **/MPa**
	1.56	0.9	20	8.0	1.0
**Bolt** **property**	**Elastic modulus** **/GPa**	**Nominal diameter** **/mm**	**Lengh** **/m**	**Pretension** **/KN**	**Bond stiffness** **/N.m** ^**-2**^
	200	20	2.4	200	10e7

**Fig 2:**
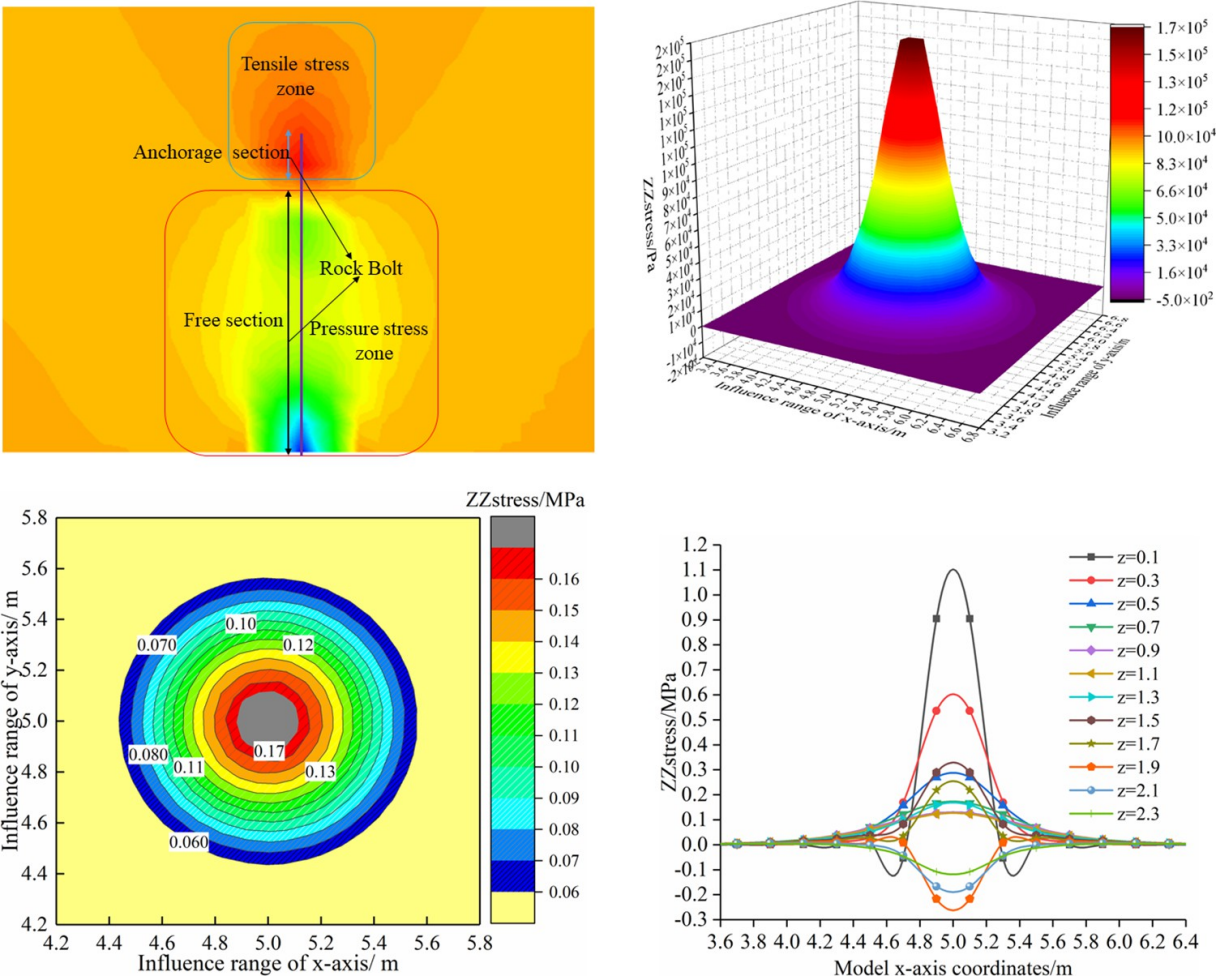
Distribution of stress field under single bolt supporting.

The stress field distribution under a single bolt support is shown (Fig 2). As shown in Fig 2(A), 2(B), 2(C) and 2(D), according to the definition of positive pressure and negative tension, the compressive stress field of a single bolt is roughly in the shape of an "apple", with the compressive stress value near the bolt tray is the largest, and the compressive stress area in the middle part is larger. A compressive stress concentration area appeared near the anchoring section. The stress field perpendicular to the bolt along the length of the bolt has an approximately "conical" distribution. The closer to the centre of the bolt, the greater the supporting stress, and the smaller the supporting range. In addition, under the action of bolt support, the maximum compressive stress of the surrounding rock is 1.13 MPa, and the maximum tensile stress is 0.28 MPa. The controlling influence angle between the compressive zone and tensile stress is also different. This is not consistent with traditional combined arch theory.

The distribution range and shape of the bolt supporting stress field are related to many factors, and are mainly divided into bolt characteristic factors, which include bolt material, bolt specification, and applied pretension and environmental factors, such as rock lithology and the development characteristics of rock mass cracks. It was necessary to quantify the influencing factors of β.

Therefore, the influence of MOE (Modulus of Elasticity) of bolt, bolt diameter, rock strength, pretension and bolt length under the action of bolt support was analysed, and then the characteristics of β were further analysed. The designed test simulation scheme is shown in Table 2.

**Table 2:** Simulation scheme.

Influence factor	1	2	3	4	5
MOE of bolt(e10 Pa)	2	4	6	8	10
bolt diameter(mm)	16	20	24	28	32
Cohesion(MPa)	0.7	1	1.3	1.6	2
Pretension (kN)	100	150	200	250	300
bolt length(m)	2.0	2.2	2.4	2.6	2.8

## 2.2 Experimental results and analysis

**Fig 3:**
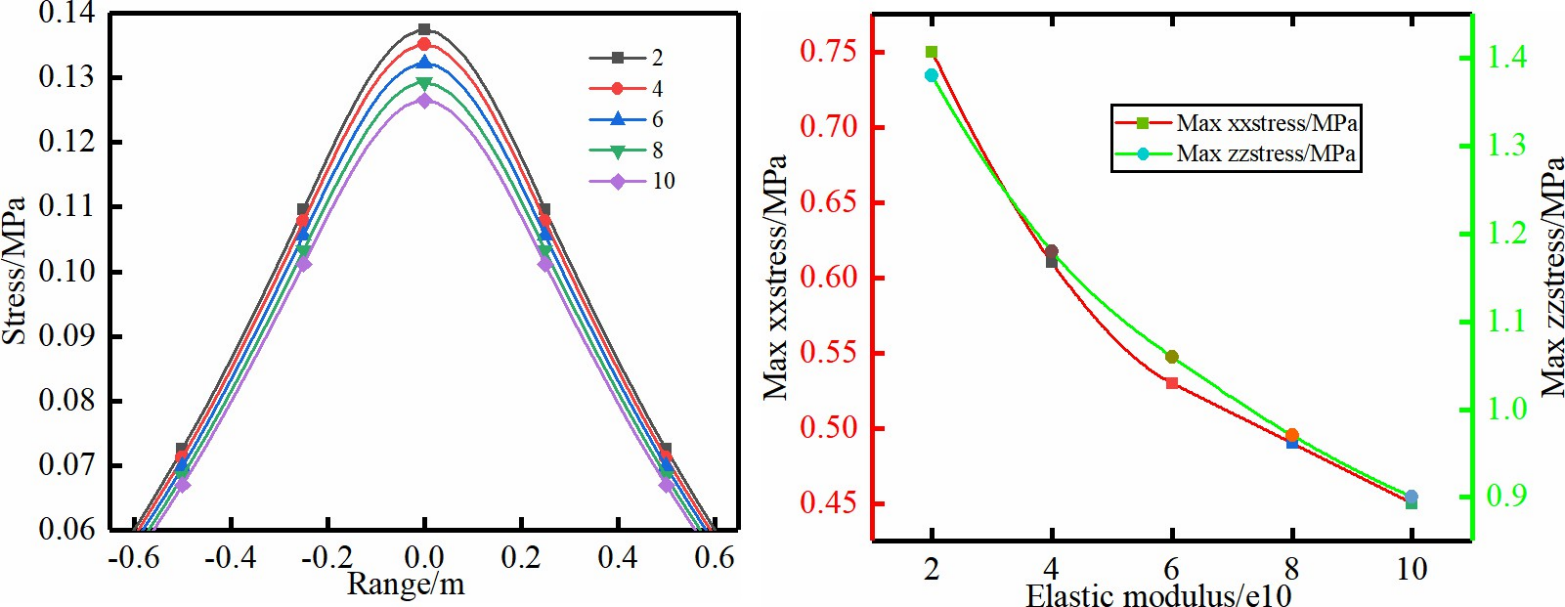
Different elastic modulus.

**Fig 4:**
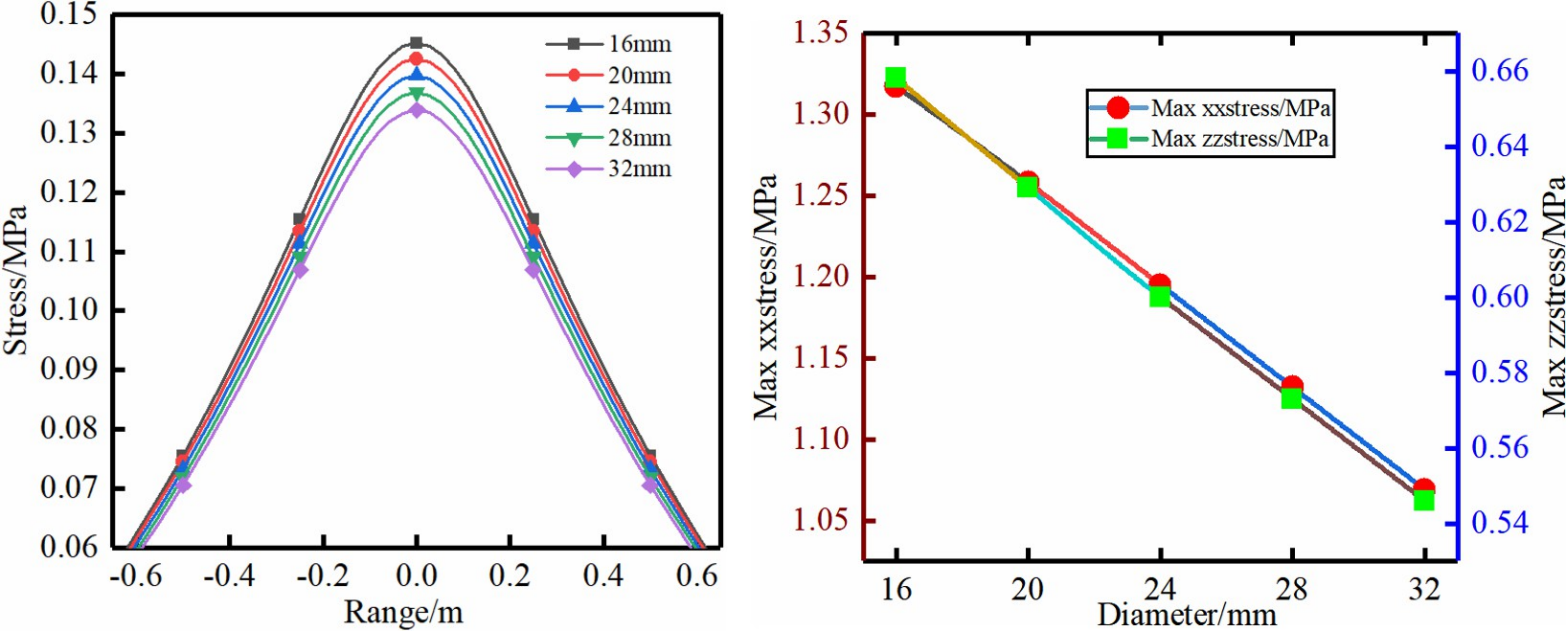
Different diameter.

**Fig 5:**
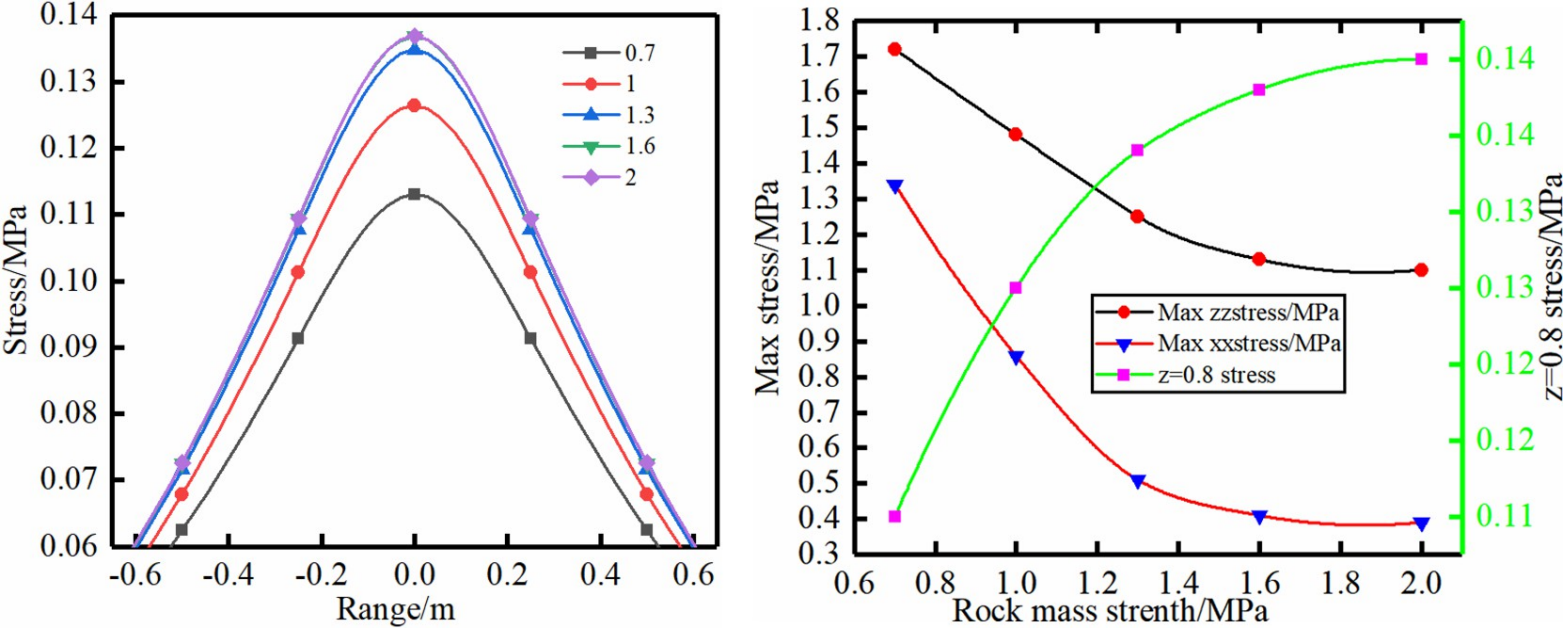
Different rock mass strength.

**Fig 6:**
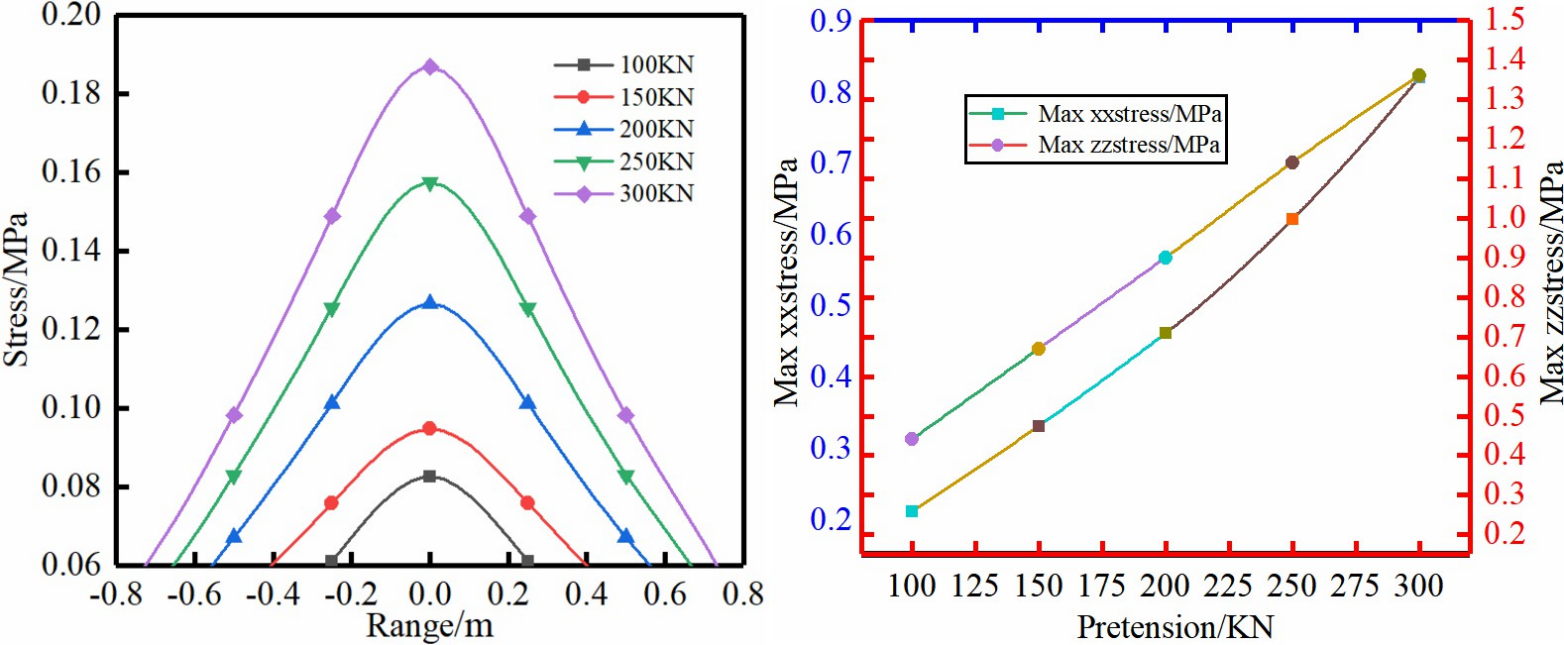
Different pretension.

**Fig 7:**
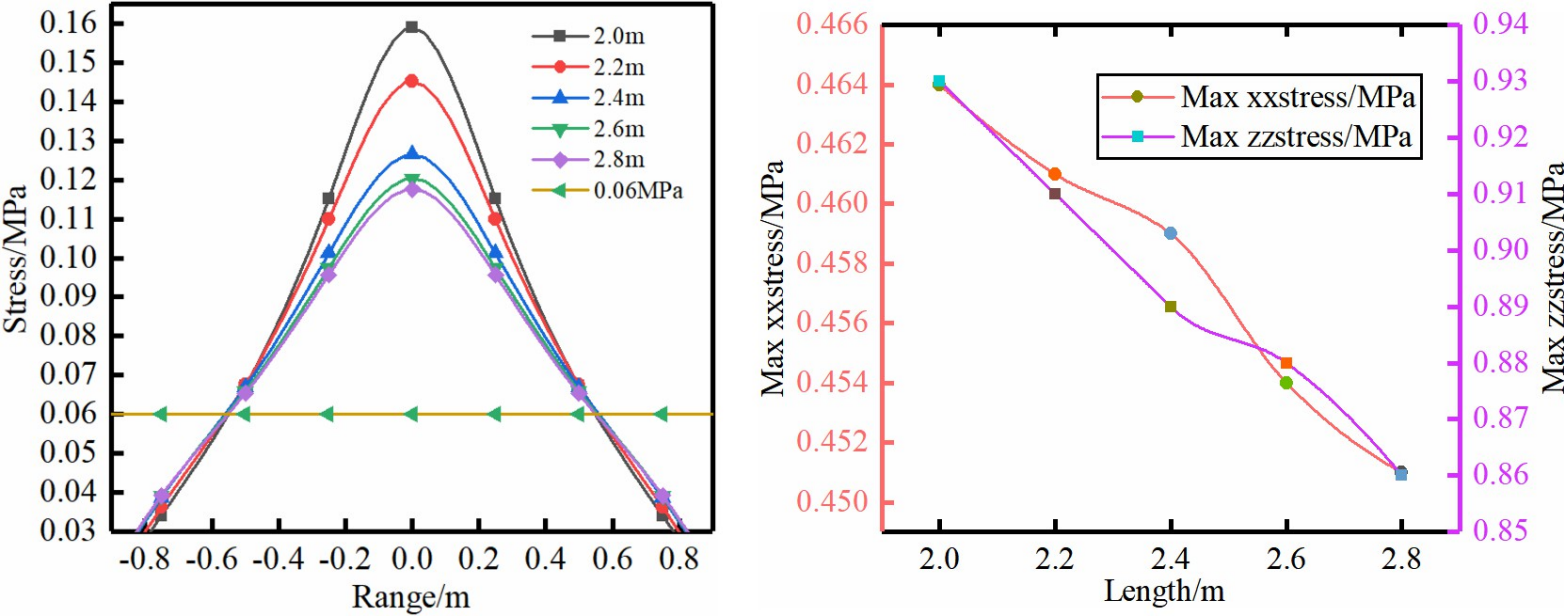
Different bolt length.

To study the effective controlling range of bolt support, the effective stress of bolt support is 30% to 60% of the yield stress based on the “Code for bolt support for coal mine roadways”. Without considering the ground stress, the effective stress of the bolt support was defined as 0.06 MPa, which is the boundary of the support-induced stress. The entire stress distributions of schemes (1)–(5) are shown in Figs 3–7. The parameter characteristics were described and analysed as follows.

Scheme (1): The stress of the bolt support is distributed symmetrically along the bolt axis (Figs 3–7). The stress curve of the model z = 0.85 m is shown in Fig 3. With the gradual increase in the elastic modulus of the bolt, the supporting range gradually decreases, and the maximum stress of the supporting stress field also gradually decreases. In addition, the maximum vertical stress and horizontal stress show a decreasing trend with the increase of the elastic modulus. The reduction range of the maximum vertical stress and the horizontal stress value reaches 35% and 40%, respectively.

Scheme (2): The diameter of the bolt should meet the maximum anchoring force without breaking. Therefore, the diameter of the bolt is chosen to be 16–32 mm. The stress distribution characteristics of the scheme have a trend similar to that of scheme (1). The reduction range of the maximum vertical stress and the horizontal stress value reaches 22% and 20.5%, respectively.

Scheme (3): Due to the existence of pores and joints, the compressive strength of rock is much greater than that of rock mass, and some even reach several times the strength of rock mass based on rock mechanics. Therefore, the cohesion in the rock mass is chosen to be 0.7–2.0 MPa. The stress distribution characteristics of the scheme have an opposite trend to schemes (1) and (2). However, the maximum support range and maximum stress change are no longer obvious when the cohesion exceeds 1.6 MPa, and the stress value gradually increases with greater cohesion in the z = 0.8 m plane.

Scheme (4): The pretension in this scheme is chosen to be 100–300 kN. The extension range of the rock mass surface compression zone shows a gradually increasing trend with the increase of the pretension, and the compressive force value also increases with the increase of the pretension. Therefore, the increase in the pretension will further squeeze and compact the surface rock mass, limit the deformation of the rock mass and improve the mechanical properties of the rock mass, and the influence range of bolt support will gradually increase.

Scheme (5): The largest influence range is not very obvious for different bolt lengths, and the stress value gradually decreases with longer bolts. The rock bolt is mainly subjected to a shearing force to prevent horizontal deformation and movement of the rock formation.

## 3. Prediction of the controlling influence angle

### 3.1 Equation of the controlling influence angle

**Fig 8:**
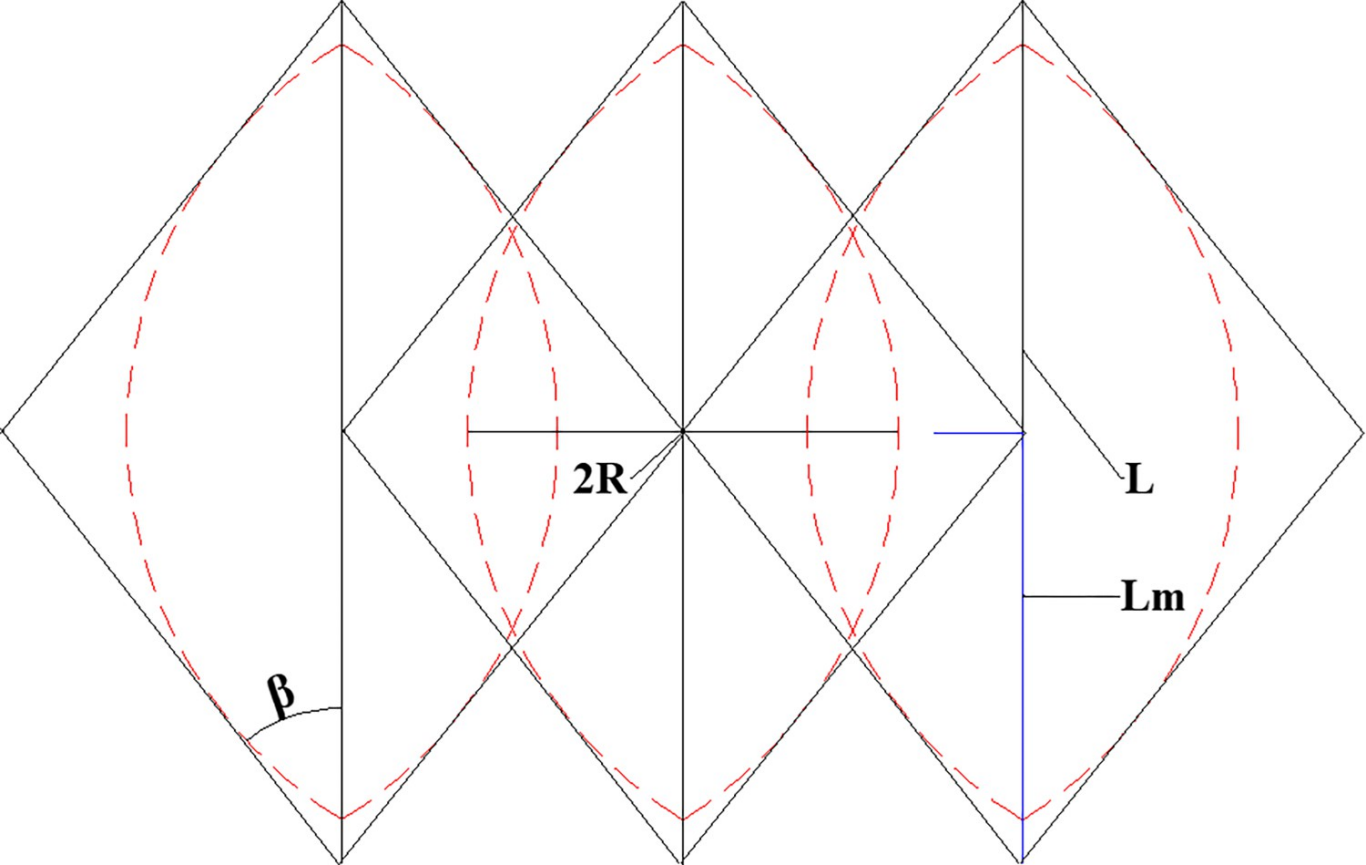
Anchor support reinforced arch diagram.

The combined arch formed by bolt support is shown (Fig 8).

Related research scholars have proposed the concept of the prestress diffusion coefficient [33]. The prestress diffusion coefficient (*k*_*d*_) is the ratio of the width of the effective compressive stress zone formed by the prestress of the bolt in the surrounding rock to the length of the bolt. This paper defines the controlling influence angle of a single bolt as *β*. Along the length of the bolt, the maximum supporting influence radius of the bolt supporting the surrounding rock is *R*, and the corresponding length is *L*_*m*_ metres from the position of the tray. Therefore, the equation for calculating the controlling influence angle (*β*) is obtained as follows:

$$\beta =\tan^{-1}\frac{R}{L_{m}}$$ (1)

**Fig 9:**
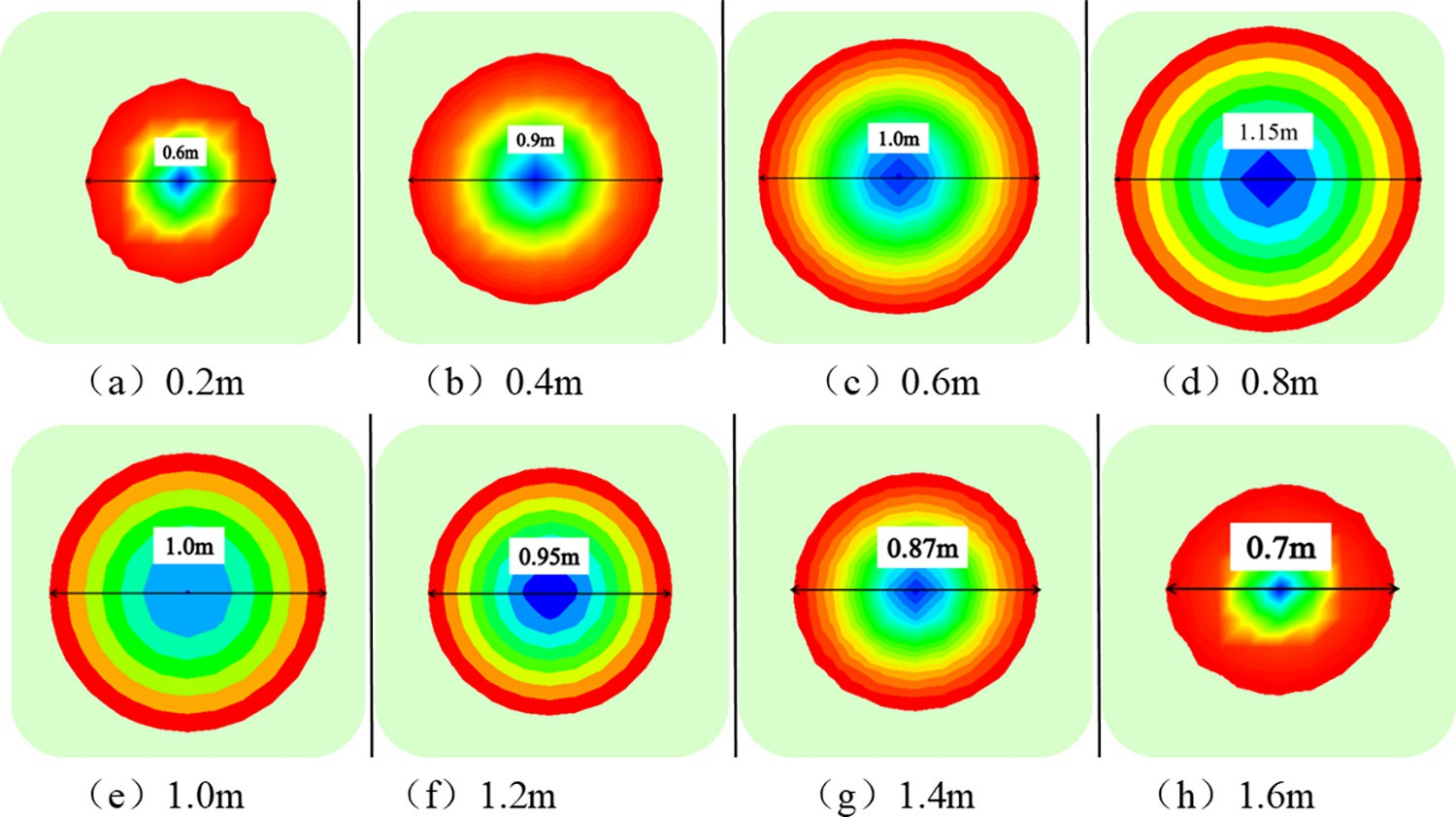
Compressive stress range diagram of different sections along the length of the bolt.

**Fig 10:**
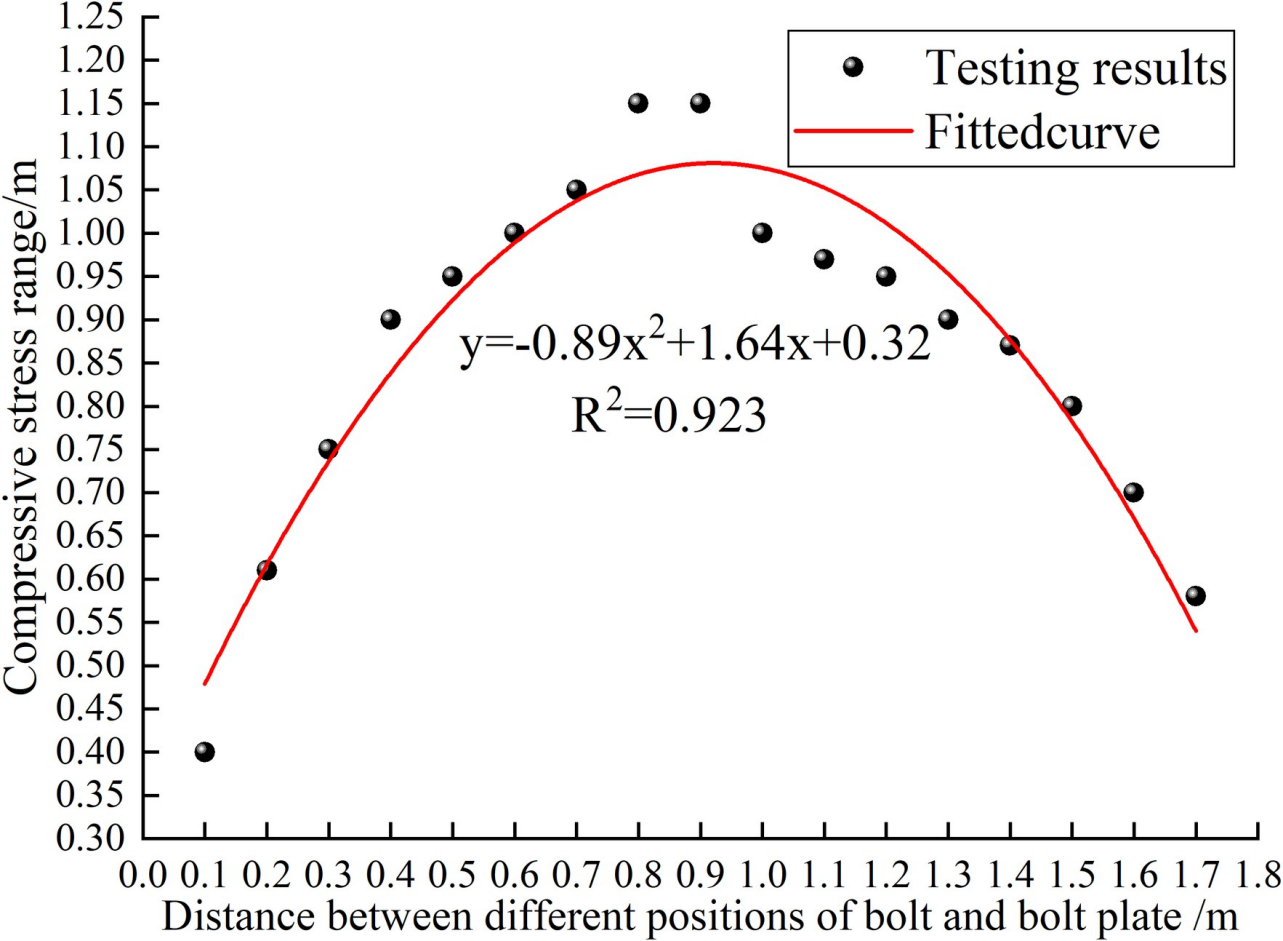
Curves of compressive stress range of different sections.

To obtain the optimal value of *L*_*m*,_ it is set at 0.2 m-1.6 m. Furthermore, the compressive stress ranges of different cross-sections are obtained (Fig 9). The results are shown (Fig 10).

As shown in Figs 9 and 10, the compressive stress range is a circle with a diameter of 0.6 m when *L*_*m*_ is 0.2 m along the axis of the bolt. Gradually moving away from the tray, the compressive stress range gradually increases. Moreover, the compressive stress range is the largest, which is a circle with a diameter of 1.19 m when *L*_*m*_ is 0.85 m. As the bolt continues to penetrate the rock formation, the compressive stress range drops to a circle with a minimum diameter of 0.7 m. Afterwards, the compressive stress gradually decreases and turns into tensile stress.

Based on the above analysis, when *L*_*m*_ is 0.85 m, half of the length of the free section of the anchor bolt, the rock bolt support has the largest range. Therefore, the value of *L*_*m*_ is fixed at 0.85 m under a constant bolt length.

### 3.2 Prediction of the controlling influence angle

#### 3.2.1 Analytical solution

**Fig 11:**
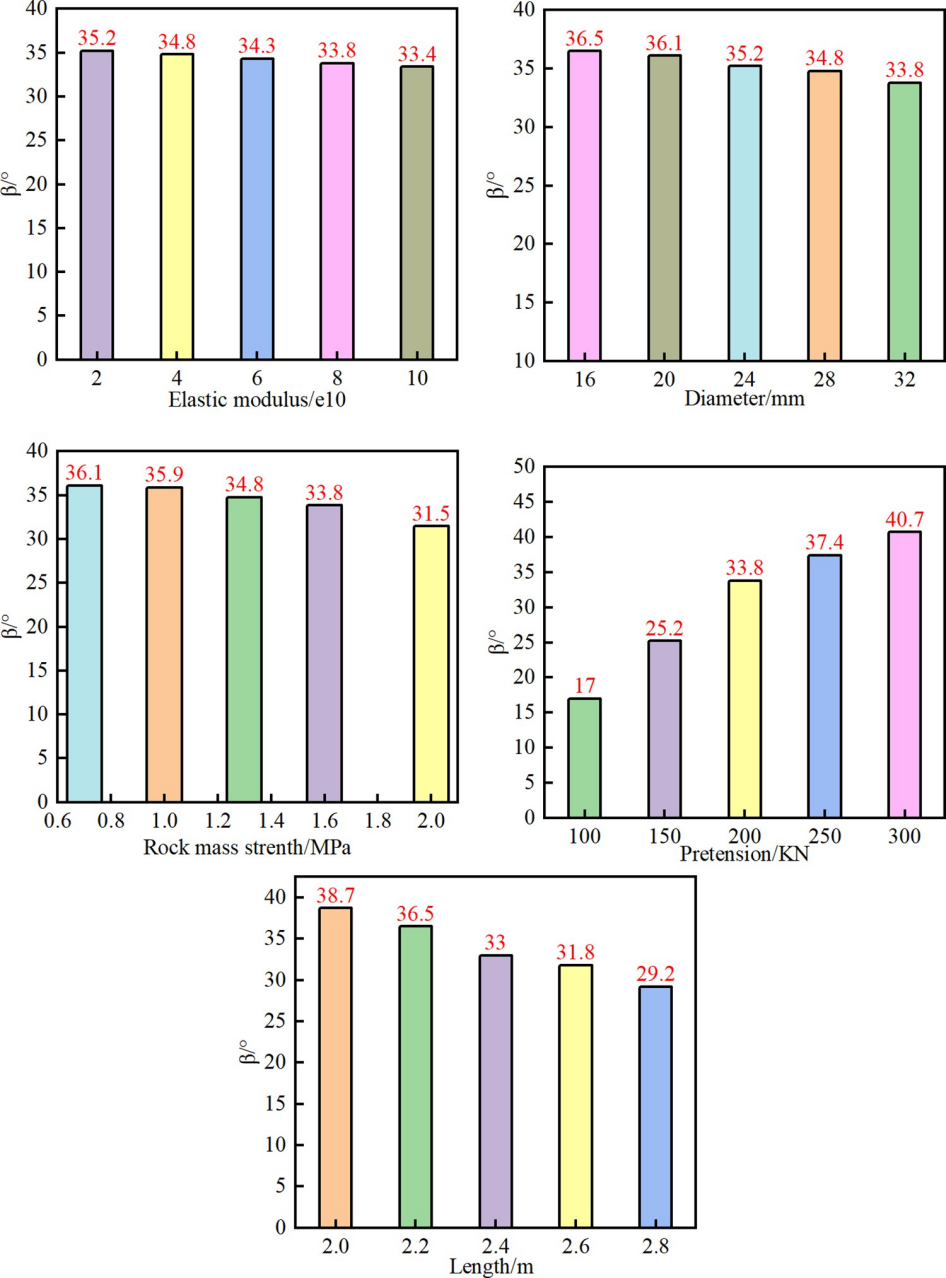
Influence angle β under different factors.

The influence range data recorded during the tests were used to analyse the influence angle characteristics of the bolt support. The influence angle law of the bolt support for five factors are shown (Fig 11).

As the elastic modulus of the bolt gradually increases, β gradually decreases, the decreasing trend is not obvious, with the lowest value of 33.4°, and the range of change is small (see Fig 11(A)).

Similarly, as the diameter of the anchor bolt gradually increased, β also gradually showed a downward trend, with the lowest value being 33.8°, which is a decrease of 8% (see Fig 11(B)).

The change of *β* can be viewed (see Fig 11(C)). The results showed that the greater the strength of the rock mass, the greater the *β* of bolt support. *β* stabilizes at approximately 36.1° when the strength increases to a certain value. When the strength of the surrounding rock is large enough, the deformation and damage of the surrounding rock mass are relatively low.

Fig 11(D) shows that the pretension has the most obvious influence on β. When the pretension reaches the maximum value of 300 kN, β reaches the maximum value of 40.7°. As a result, providing a larger pretension has the best support effect in soft coal seams.

Fig 11(E) shows β under different lengths of rock bolts. With the gradual increase in the length of the bolt, the change in the trend of the controlling influence range is smaller. According to the definition of the controlling influence angle in this article, the longer the bolt length, the larger the *Lm*, and the smaller the *β* value. When the bolt length is 2.8 m, β drops to the lowest value of 29.2°.

#### 3.2.2 Synthetic weight analysis of the controlling influence angle

The curve fitting results of the controlling influence angle are shown in Table 3. The FAHP-entropy weight method is used to determine the weight of the bolt controlling influence angle β with the following five factors: the elastic modulus of the anchor (A), the diameter of the anchor bolt (B), the strength of the rock mass (C), pretension (D) and anchor bolt length (E). This method combines subjective evaluation and objective evaluation, which mitigates errors caused by the artificial determination of the relative importance of each factor and the extreme value of the entropy weight method by AHP. In addition, obtaining the weight result of each factor more accurately is an added advantage.

**Table 3:** Curve fitting equation of β under different factors.

Influence factor	Fitting equation	variance
Elastic modulus of bolt	Y_1_ = -0.0006x^2^–0.2224x + 35.665	R^2^ = 1
The bolt diameter	Y_2_ = -0.0003x^4^ + 0.028x^3^–0.9898x^2^ + 15.07x - 46.84	R^2^ = 0.99
Cohesion	Y_3_ = -12.297x^4^ + 66.876x^3^–133.9x^2^ + 120.28x - 7.1153	R^2^ = 0.9899
Pretension	Y_4_ = 0.0156x^2^–1.6616x + 75.67	R^2^ = 0.9958
The bolt length	Y_5_ = -186.19x^4^ + 1787.3x^3^–6398.6x^2^ + 10112x - 5910.1	R^2^ = 0.987

First, the impact of each factor was analysed, and a fuzzy judgement matrix by the fuzzy analytic hierarchy process was constructed in Table 4. Then, a consistent matrix can be obtained after fuzzy transformation.

**Table 4:** Judgment matrix of each factor evaluation index.

	A	B	C	D	E
**A**	0.38	0.38	0.48	0.53	0.43
**B**	0.38	0.38	0.48	0.53	0.43
**C**	0.48	0.48	0.58	0.63	0.53
**D**	0.53	0.53	0.63	0.68	0.58
**E**	0.43	0.43	0.53	0.58	0.48

The weight *w*_*i*_ can be denoted by

$$w_{i}=\frac{1}{n}-\frac{1}{2a}+\frac{1}{na}\sum_{k=1}^{n}r_{ik}$$ (2)

where *n* is the order of the matrix and can be expressed by Eq (21) [34]:

$$a=\frac{n‐1}{2}$$ (3)

Determine the weight of each Factor *W*_1_ by Eq (4):

$$W_{1}=(0.17,0.17,0.22,0.245,0.195)$$ (4)

The nonfuzzy matrix can be obtained with the defuzzification algorithm using Eq (5), as shown in Table 5 [35]:

$$R_{\mathrm{ij}}=3^{5(r_{\mathrm{ij}}-0.5)}$$ (5)

**Table 5:** Non fuzzy judgment matrix of each factor.

	A	B	C	D	E
**A**	1	1	0.333	0.192	0.577
**B**	1	1	0.333	0.192	0.577
**C**	3	3	1	0.577	1.732
**D**	5.196	5.196	1.732	1	3
**E**	1.732	1.732	0.577	0.333	1

Determine the information entropy and entropy weight of the evaluation index and obtain the weight *W*_*2*_ of each factor

$$W_{2}=(0.02,0.02,0.24,0.64,0.08)$$ (6)

The results *W*_*1*_ and *W*_*2*_ are analysed by the weighted average method and substituted into the equation [36].

The final comprehensive weight W can obtain:

W=(0.06,0.06,0.27,0.46,0.15) (7)

**Fig 12:**
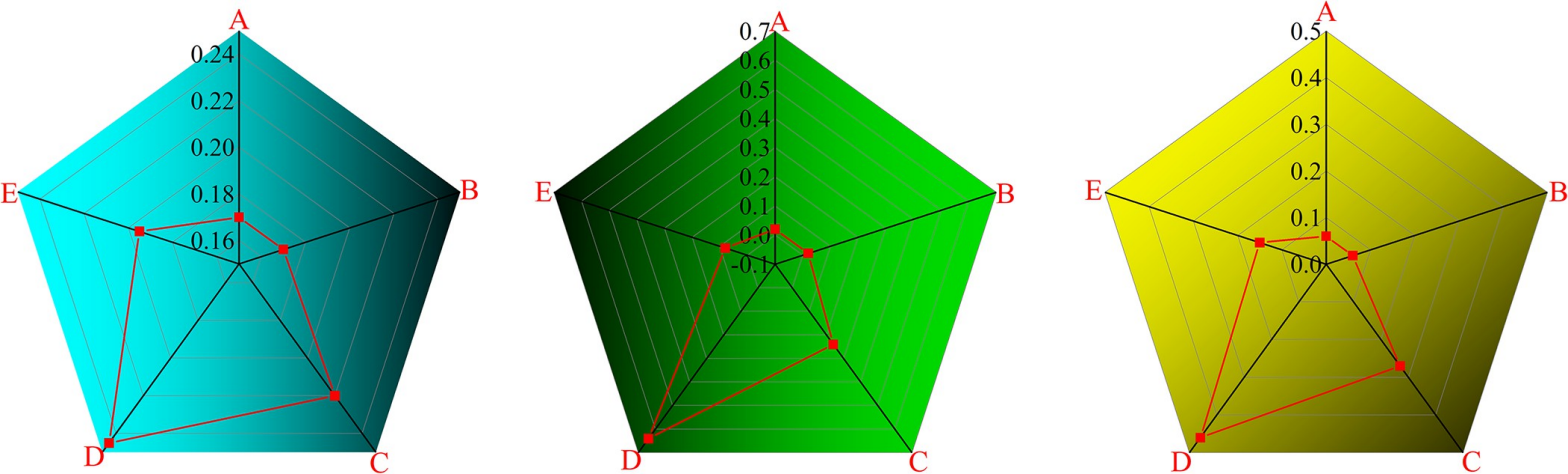
Weighted radar chart.

The results are shown in Table 6 after sorting out the weight of each factor of influence of the angle of bolt control. The obtained subjective weight and objective weight data are imported into the radar chart to obtain the weight of each factor (Fig 12).

**Table 6:** Sort table of importance.

Factor	AHP-Subjective weight	Entropy weight—Objective weight	Comprehensive weights	Rank
D	24.5%	64%	46%	1
C	22%	24%	27%	2
E	19.5%	8%	15%	3
A	17%	2%	6%	4
B	17%	2%	6%	4

As shown in Table 6 and Fig 12, the factor of rock bolt pretension was ranked first in terms of the importance on the influence angle irrespective of using the AHP method or using the entropy weight method, and it accounted for 24.5% and 64%, respectively. The weight of the bolt elastic modulus was obtained and had a similar trend as the bolt diameter. Both of them had less effect on the influence angle and accounted for 6%. Surrounding rock mass strength was also an important indicator of the influence angle, in which the comprehensive weight accounted for 27%, and the bolt length index was ranked third in terms of the importance of the influence angle, in which the comprehensive weights accounted for 15%.

In conclusion, the comprehensive weights and the rank that affected the influence angle β of bolt were given, and the order of comprehensive weights was D>C>E>A = B. It was similar to the order of AHP-Subjective weight and Entropy Objective weight.

Let $Y={\left(Y_{1},Y_{2},Y_{3},Y_{4},Y_{5}\right)}^{T}$, $W=(W_{A},W_{B},W_{C},W_{D},W_{E})$

The prediction matrix of the influence angle can be obtained when the weights of each factor with the fitting equation are combined as follows:

$$\beta =(\begin{array}{ccccc} W_{A}Y_{1} & 0 & 0 & 0 & 0\\ 0 & W_{B}Y_{2} & 0 & 0 & 0\\ 0 & 0 & W_{C}Y_{3} & 0 & 0\\ 0 & 0 & 0 & W_{D}Y_{4} & 0\\ 0 & 0 & 0 & 0 & W_{E}Y_{5} \end{array})$$ (8)

The prediction model of *β* can be obtained as follows:

$$\beta =W_{A}Y_{1}+W_{B}Y_{2}+W_{C}Y_{3}+W_{D}Y_{4}+W_{E}Y_{5}$$ (9)

## 4.Verification and comparison

To validate the proposed model, the analytical predictions from the model were compared with the existing result, in which the value of *β* was 45°. The thickness *b* of the composite arch and the load-bearing force of the composite arch along the axial unit length of the roadway can be seen based on reference [37]:

$$b=\frac{l\tan\beta -D}{\tan\beta }$$ (10)

where *l* is the effective length and *D* is the interval and row spacing of bolt.


$$Q=\frac{T_{S}}{D^{2}}\frac{1+\sin\varphi_{s}}{1-\sin\varphi_{s}}b$$ (11)

The 103 face transportation tunnel of Xinjiang Dabei Mining is considered as the research background. The original support scheme is calculated according to the control influence angle *β* of 45°. The surrounding rock parameters *C*, *Φ*_*s*_, pretension *T*_*s*_ and bolt length *L* of the 103 face transportation tunnel were 1 MPa, 30°, 100 kN and 2.4m, and the row spacing between bolts *D* and the elastic modulus of the bolt *E*_*a*_ were 0.9 m and 20 GPa. The thickness and bearing force of the composite arch formed by the bolt support are obtained as follow:

$$b_{1}=1.5m$$ (12)

$$Q_{1}=5.55\times 10^{5}Pa$$ (13)

According to the prediction model of *β* from the paper, the control influence angle is obtained by substituting each parameter into the model.


$$\beta =WY=0.06Y_{1}+0.06Y_{2}+0.27Y_{3}+0.46Y_{4}+0.15Y_{5}$$ (14)

Among them, *Y*_*1*_, *Y*_*2*_, *Y*_*3*_, *Y*_*4*_ and *Y*_*5*_ can be obtained according to the *β* fitting formula of different factors in Table 3.

Based on the Eq (14), the specific value of *β* is 49°.

Substituting *β* into the resultant bearing energy equation of the composite arch, the thickness and bearing capacity of the composite arch formed by roadway bolting support in this mine are obtained as follows:

$$b_{2}=1.62m$$ (15)

$$Q_{2}=6.0\times 10^{5}Pa$$ (16)

**Fig 13:**
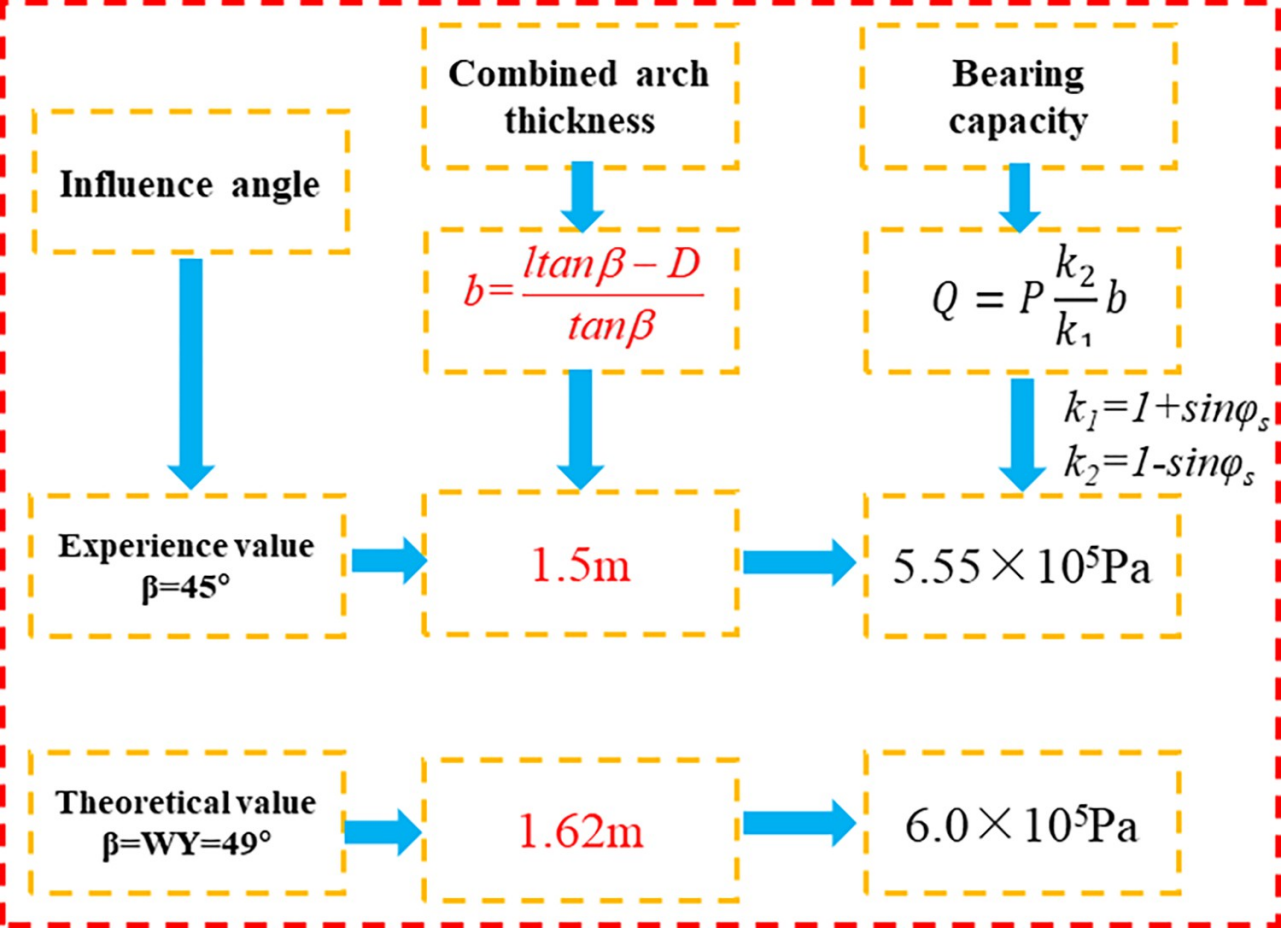
Comparison of empirical values and predication model.

The prediction model is verified by comparisons with empirical results of the influence angle β, and the prediction β from the analytical model is 49°, which is greater than 45° (Fig 13). By comparing the above analysis results, it can be seen that the actual load-bearing force of the roadway bolt support is 6.07×10^5^Pa, under the geological conditions of the mine, which is larger than the load-bearing force when the control influence angle is 45° based on experience. This proves that the support parameter design based on the original scheme is more reasonable, and can meet the actual production safety and quality requirements and ensure the stability of the surrounding rock of the roadway.

## 5. Conclusions

1. The supporting-induced stress field of the prestress bolt is roughly in an "Apple shape" distribution. There is a large supporting stress near the bolt and gradually decreases with the large distance from the centre of the rock bolt. Moreover, the supporting range decreases near the centre of the bolt.
2. The distribution characteristics of the stress field under bolt support can be obtained and are related to the factors of the bolt and the environment. The larger the elastic modulus and the diameter of the bolt, the smaller the maximum stress of the bolt support and the range of influence of the support. The increase in the pretension gradually changes the stress field of the support from continuous to the whole, and the maximum stress and range of the support are significantly increased. The shorter the bolt length is, the greater the active supporting force. Increasing the bolt length mainly prevents the horizontal deformation and movement of the rock formation.
3. The effect of several key parameters on bolt support was presented. Based on the numerical simulation and results that obtained during bolt support, the β of the bolt is not stable, and an analytical model was proposed to predict the law of influence angle under bolt support. As the elastic modulus, the diameter and length of the bolt increase, the influence angle decreases. Conversely, as the surrounding rock strength increases or as the pretension increases, the influence angle of a bolt increases.
4. The prediction model is verified by comparisons with traditional results of the influence angle β under the Dabei Mining 103 face transportation tunnel, and the prediction β from the analytical model is 49°, which is greater than 45°. The rationale behind the original support design scheme can be verified.

## References

[1] Skrzypkowski K. An Experimental Investigation into the Stress-Strain Characteristic under Static and Quasi-Static Loading for Partially Embedded Rock Bolts. Energies, 2021, 14(5):1483. doi: 10.3390/en14051483

[2] Kang HP. Seventy years development and prospects of strata control technologies for coal mine roadways in China. Chinese Journal of Rock Mechanics and Engineering.2021,40(1):1–30.

[3] Kang HP, Jiang PF, Cai JF. Test and analysis on stress fields caused by rock bolting. Journal of China Coal Society.2014,39(8):1521–1529.

[4] Lin J, Wang Y, Yang JH, Wang ZS. Analogue simulation of supporting stress field characteristic of single anchored bolt under different working loads. Coal Mining Technology. 2015,20(05):87–92+22.

[5] Lu SL, Tang L, Yang XN. Anchoring force and technology of bolt[M]. Beijing: Coal Industry Press.1998: 8–12.

[6] IME. Seventh progress report of investigation into causes of falls and accidents due to falls-improvement of working conditions by controlled transference of roof load. Transactions of the Institution of Mining Engineers. 1949, 108(11): 489–504.

[7] Zhang ZX, Xu Y, Kulatilake P, Huang X. Physical model test and numerical analysis on the behavior of stratified rock masses during underground excavation. International Journal of Rock Mechanics & Mining Sciences. 2012, 49(none):134–147. doi: 10.1016/j.ijrmms.2011.11.001

[8] Dancygier A.N., Karinski Y.S., Chacha A. A model to assess the response of an arched roof of a lined tunnel. Tunnelling and Underground Space Technology. 2016, 56: 211–225. 10.1016/j.tust.2016.03.009.

[9] Zhang Z, Kang HP, Wang JH. Pre-tensioned stress coordination function analysis of bolt-cable anchor support in coal roadway. Journal of China Coal Society. 2010,35(06):881–886.

[10] Novoselac, Kozak S, Dražan, Ergić, et al. Influence of stress gradients on bolted joint fatigue behaviour under different preloads and cyclic loads ratio. Structural integrity and life. 2014.

[11] Kang HP, Jiang PF, Wang Z, Liu Q, Yang J, Gao F, et al. Roadway strata control technology by means of bolting-modification-destressing in synergy in 1000 m deep coal mines. Journal of China Coal Society. 2020,45(03):845–864.

[12] Yu Y, Wang X, Bai J, Zhang L, Xia H. Deformation mechanism and stability control of roadway surrounding rock with compound roof: research and applications. Energies. 2020, 13(6):1350. 10.3390/en13061350.

[13] Ji YM. Study of the infrared temperature field and stress field for a bolted rock. Journal of Experimental Mechanics. 2012,27(02):244–248.

[14] Zhang YD. Study on bearing characteristic of composite bolt-rock bearing structure and its application in roadway bolting design. China University of Mining and Technology,2013.

[15] Wang XQ, Kang HP, Gao FQ, Lou JF, Li JZ, Yang L. Analysis of pressure arch formation and rock bolt function in gravel bolting. Journal of China Coal Society. 2021,46(09):2865–2873.

[16] Li JZ, Kang HP, Gao FQ. Analysis of bolt supporting-induced stress field and bolt support effect under in-situ stress field. Journal of China Coal Society. 2020,45(S1):99–109.

[17] Liu DJ, Zuo JP, Liu HY, Hong ZJ, Wen JH, ShiY. Development and present situation of support theory and technology in coal mine roadway in China. Journal of Mining Science and Technology. 2020,5(01):22–33.

[18] Cai Y, Tetsuro Esaki, Jiang YJ. A rock bolt and rock mass interaction model. International Journal of Rock Mechanics and Mining Sciences.2004,41(7): 1055–1067. 10.1016/j.ijrmms.2004.04.005.

[19] Gao S, Zhang G, Gao G. Application analysis of bolt-mesh-anchor support in surrounding rock of coal roadway under complex stress field. Safety in Coal Mines. 2018,49(01):168–171.

[20] Gao FQ, Stead D, Kang HP, Wu YZ. Discrete element modelling of deformation and damage of a roadway driven along an unstable goaf—A case study. International Journal of Coal Geology. 2014, 127:100–110. 10.1016/j.coal.2014.02.010.

[21] Kang HP, Yang JH, Meng XZ. Tests and analysis of mechanical behaviours of rock bolt components for China′s coal mine roadways. Journal of Rock Mechanics and Geotechnical Engineering. 2015,7(1):14–26. 10.1016/j.jrmge.2014.12.002.

[22] Zhao YJ, Yang Y, He L, Wang W, Lei M, Yan XN. Numerical simulation of reinforcement arch theory of high pre-stress anchor bolt. Safety in Coal Mines. 2014,45(09):47–50.

[23] Li XW, Naj Aziz, Ali Mirzaghorbanali, Jan Nemcik. Behavior of Fiber Glass Bolts, Rock Bolts and Cable Bolts in Shear. Rock Mechanics and Rock Engineering. 2016,49(7):2723–2735. 10.1007/s00603-015-0907-7.

[24] Chen YL, Teng JY, Rana Ammad, Zhang K. Experimental Study of Bolt-Anchoring Mechanism for Bedded Rock Mass. International Journal of Geomechanics. 2020,20(4). 10.1061/(ASCE)GM.1943-5622.0001561.

[25] He L, An XM, Zhao XB, Zhao ZY, Zhao J. Development of a Unified Rock Bolt Model in Discontinuous Deformation Analysis. Rock Mechanics and Rock Engineering. 2018, 51(3):827–847. 10.1007/s00603-017-1341-9.

[26] Wei W, Jiang Q, Peng J. New Rock Bolt Model and Numerical Implementation in Numerical Manifold Method. International Journal of Geomechanics. 2017, 17(5): E4016004.1–E4016004.12. 10.1061/(ASCE)GM.1943-5622.0000669.

[27] Chen JH, Liu P, Liu L, Zeng BQ, Zhao HB, Zhang C, et al. Anchorage performance of a modified cable anchor subjected to different joint opening conditions. Construction and Building Materials. 2022, 336: 1–12. 10.1016/j.conbuildmat.2022.127558.

[28] Chen JH, Zeng BQ, Liu L, Tao KM, Zhao HB, Zhang C, et al. Investigating the anchorage performance of full-grouted anchor bolts with a modified numerical simulation method. Engineering Failure Analysis. 2022, 141: 1–14. 10.1016/j.engfailanal.2022.106640.

[29] Zhang GC, He FL. Deformation failure mechanism of high stress deep soft roadway and its control. Journal of Mining & Safety Engineering. 2015,32(4):571–577.

[30] Song HW, Mu BS. Research on load capability and rational thickness of a reinforced broken rock arch. Journal of China University of Mining & Technology. 1997,26(2):33–35.

[31] Meng QB, Qian W, Han LJ, Mei FQ, Feng W, Zhou X. Formation mechanism of arch structure balanced by double-layer anchor in extremely weak strata. Journal of Mining & Safety Engineering. 2019,36(4):650–656.

[32] Li YM, Zhao CX, Liu ZH. Research on layered evolution law of surrounding rock bearing layers and strength analysis of “layer-double arch” bearing structure. Chinese Journal of Rock Mechanics and Engineering. 2020,39(2):217–227.

[33] Kang HP, Jiang TM, Gao FQ. Effect of pretensioned stress to rock bolting. Journal of China Coal Society. 2007(07):680–685.

[34] Wang ZH. Study on comprehensive index weight of working face based on combined weights of AHP-Entropy. Inner Mongolia Coal Economy,2019(08):3–5.

[35] Wang M. Research on safety comprehensive evaluation method based on entropy weight fuzzy logarithm first programming theory. University of science and technology of China. 2017.

[36] Wang FQ, Ma SY, Zhao H, Liu PH. Fuzzy comprehensive evaluation of water circulation health in Beijing-Tianjin-Hebei region based on combined weights of AHP and Entropy. South-to-North Water Transfers and Water Science &Technology. 2021,19(01):67–74.

[37] Yu WJ, Gao Q, Zhu CQ. Study of strength theory and application of overlap arch beating body for deep soft surrounding rock. Chinese Journal of Rock Mechanics and Engineering. 2010, 29(10):2134–2142.

